# Three-dimensional clinical handheld photoacoustic/ultrasound scanner

**DOI:** 10.1016/j.pacs.2020.100173

**Published:** 2020-03-11

**Authors:** Changyeop Lee, Wonseok Choi, Jeesu Kim, Chulhong Kim

**Affiliations:** a Department of Mechanical Engineering, Pohang University of Science and Technology (POSTECH), 37673, Pohang, Republic of Korea; b Department of Electrical Engineering, Pohang University of Science and Technology (POSTECH), 37673, Pohang, Republic of Korea; c Department of Creative IT Engineering, Pohang University of Science and Technology (POSTECH), 37673, Pohang, Republic of Korea; d Departments of Creative IT Engineering, Mechanical Engineering, and Electrical Engineering, Pohang University of Science and Technology (POSTECH), 37673, Pohang, Republic of Korea

**Keywords:** Clinical translation, Handheld scanner, Photoacoustic, Scotch yoke mechanism, Three-dimensional imaging

## Abstract

Clinical 2D photoacoustic (PA) imaging can be easily implemented in a traditional ultrasound (US) system. However, 3D PA imaging is still preferable because 2D B-mode PA/US imaging suffers from low reproducibility and high-operator dependency. Here, we demonstrate a compact clinical handheld 3D PA/US scanner using an 1D linear array US transducer combined with a mechanical scanning stage working via a Scotch yoke mechanism. The entire scanner measures just 100 × 80 × 100 mm^3^ and weighs only 950 g, so it can easily be operated by hand. Blood vessels and hemoglobin oxygen saturation images of different parts of the human body (e.g., neck, wrist, thigh, and instep) have been successfully acquired. The system can potentially be used for clinical applications in fields such as oncology, dermatology, nephrology, and internal medicine.

## 1 Introduction

Photoacoustic (PA) imaging, which utilize ultrasound (US) signals from objects that have absorbed light, has become a popular medical imaging technique. When a tissue region of interest (ROI) is illuminated with short-pulsed laser light, US signals are generated by the slight thermoelastic expansions resulting from the absorption of each pulse. PA images are created by receiving the US signals with a US transducer and using beamforming techniques to reconstruct the signals into images.

Photoacoustic imaging (PAI, also called optoacoustic imaging) can map the distribution of endogenous chromophores such as oxy-hemoglobin, deoxy-hemoglobin, lipids, water, and melanin [1,2]. Furthermore, by providing functional information (e.g., total hemoglobin concentration and hemoglobin oxygen saturation (sO_2_)), PAI can aid in diagnosing diseases such as cancers and peripheral diseases [[3], [4], [5], [6], [7]]. In addition, PAI can be readily combined with widely-used US imaging on a single platform [[8], [9], [10]], creating a dual-modality PA/US imaging system that can simultaneously supply multi-parametric morphological, physiological, and molecular information [[11], [12], [13], [14], [15], [16], [17]].

In most cases, PAI is performed in 2D to provide a cross-sectional image, but the accompanying low reproducibility and high operator dependence are major problems with this approach [18]. For these reasons, automatic 3D imaging can be a promising alternative, and it is typically conducted with either a 2D array of US transducers (e.g., matrix or hemispherical) or a 1D array of US transducers (e.g., linear, convex, phased, curved, or circular) with mechanical scanning [[19], [20], [21]]. While 2D array US transducers offer volume images directly, their increased number of channels and associated electronics make the overall system very complex and expensive [22,23]. On the other hand, 1D array US transducers with mechanical scanning require fewer channels and are less complex than 2D array systems, and still provide acceptable volumetric image quality [24]. However, most mechanical scanners for 3D PA imaging are heavy and bulky, and hence not suitable for handheld operation [25,26]. One commercial 3D mechanical handheld US probe, as known as a “wobbler”, has been considered as a solution for developing 3D PA handheld scanners [27]. However, it is ultimately not suitable for PAI because the transducer rotates in a convex manner about the center axis, and thus co-axiality between the laser incidence and image planes is not ensured, resulting in low signal-to-noise ratios (SNRs). Furthermore, PA signals propagate spherically in a 3D space, and the convex scanning pattern is not adequate for receiving them.

In this paper, we demonstrate a new type of 3D clinical handheld PA/US scanner that uses a 1D linear array US transducer and a mechanical scanner. The handheld scanner is compact (100 ×  80 × 100 mm^3^) and light enough (950 g) for handheld image formation, because it uses a Scotch yoke mechanism to convert rotation into linear reciprocating movement. To confirm the device's suitability for clinical environments, we conducted multi-wavelength PAI and successfully obtained PA images of various body parts such as the neck, wrist, thigh, and instep. In addition, we performed PA sO_2_ imaging on a foot, using a spectral unmixing technique. Based on the results, we expect that the newly developed 3D handheld PA/US scanning system can be greatly useful for diagnosis and post-operative examinations of various clinical conditions of interest such as melanomas, free flaps, kidney failures, and diabetic feet.

## 2 Materials and methods

### 2.1 3D clinical handheld PA/US imaging system and scanner

**Fig. 1:**
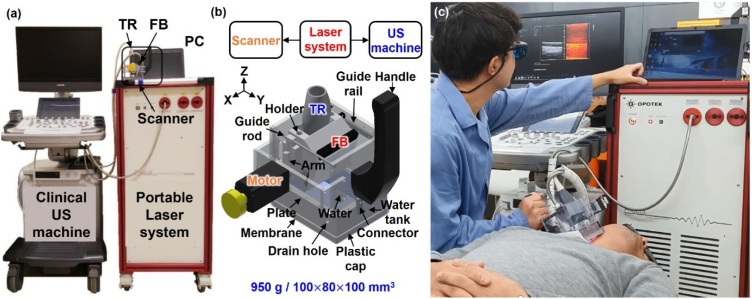
(a) Photograph of the clinical 3D PA/US imaging system. (b) Schematic of the 3D handheld PA/US scanner. (c) Photograph of the in-vivo imaging (see Supplementary Video S1). PA, photoacoustic; US, ultrasound; PC, personal computer; TR, ultrasound transducer; and FB, fiber bundle.

The clinical 3D PA/US imaging system is represented in Fig. 1a. The system consists of the programmable PA/US imaging system [28] and the 3D clinical handheld PA/US scanner. The US system is composed of a US machine (EC-12R, Alpinion Medical Systems, Republic of Korea) with 64 receiving channels and a 128-element linear array US transducer (L3-12, Alpinion Medical Systems, Republic of Korea) with a center frequency of 8.5 MHz and a fractional bandwidth of 62 % at -6 dB. While it is helpful to use broadband transducer in the context of PA imaging, we used a conventional US platform and a commercialized linear array US transducer to develop a clinically-translatable PA/US scanner. The tunable pulsed laser system (Phocus Mobile, OPOTEK, USA) is used at a pulse repetition frequency (PRF) of 10 Hz, where the wavelengths can be tuned between every pulse by the fast tuning function, and the pulsed light beam is delivered through fiber bundles (TFO-VIS100SL46-2000-F, TAIHAN FIBEROPTICS, Republic of Korea), which is linearly distributed at the output. Fig. 1b is a drawing of the handheld scanner, containing a guide rod, a holder, an arm, a geared motor, a water tank, a membrane, connectors, a plastic cap, and a handle, respectively. The guide rod minimizes shaking and tilting of the parts linked to the motor when the holder moves. The holder integrates the US transducer and the fiber bundles at 15° to optimize light transmission. The arm is connected to the holder so that the holder is driven in the elevational direction with cosine component of the angular motion by the Scotch yoke mechanism, which is a reciprocating mechanism that converts rotational motion into linear motion. Thus, the trajectory of the end of the motor arm with respect to time directly follows a simple harmonic motion because of the rotational movement of the motor with a constant angular velocity. The motor (CRK523PBK-T30, INA ORIENTAL MOTOR, Japan) weighs 170 g, and produces a 0.5 N ∙ m torque that rotates the arm. The water in the tank efficiently transmits US signals, and the 3 cm thick tank body serves as a stand-off that allows direct laser illumination to the imaging plane of the transducer [29]. At the top and bottom of the water tank, two 0.2 mm thick polyvinyl chloride (PVC) membranes seal in the water tank and provide acoustic coupling with the imaged object. The connectors facilitate convenient attachment and detachment of the water tank. The plastic cap protects imaging volunteer's skin from any sharp edge of the scanner. The handle allows an operator to hold and position the scanner conveniently. The entire handheld scanner can be positioned in any direction without spilling the water. The handheld scanner measures 100 ,  80 , and 100 mm along the x, y, and z axes, respectively. It weighs 950 g, and its field of view (FOV) is 38.4 ×40.0 mm^2^ along the x and y axes, respectively. Fig. 1c shows an operational procedure of an *in vivo* human imaging, which is also shown in Supplementary Video S1.

**Fig. 2:**
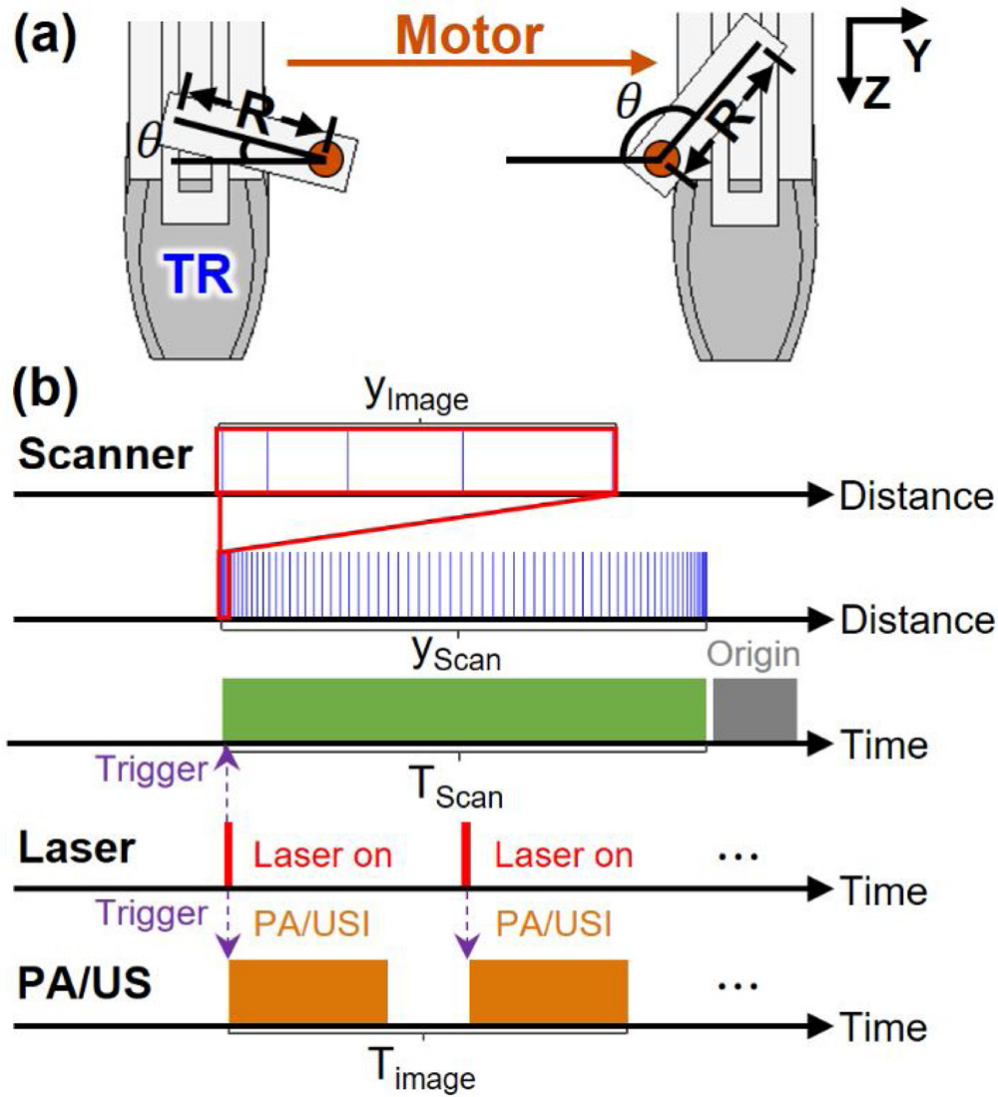
Schematics of (a) PA/US image acquisition procedure and (b) operating sequences. PA, photoacoustic; US, ultrasound; PA/USI, photoacoustic and ultrasound imaging; yImage, one frame scanning range; yScan, full scanning range; TScan, full scanning time; and TImage, imaging time.

Fig. 2 illustrates the PA/US image acquisition procedure and operating sequences. The scanner is positioned over a ROI and then moves in the elevational direction (Fig. 2a) to reconstruct a 3D PA/US image, following the operating sequences shown in Fig. 2b. After the US machine receives the trigger signal from the laser system, PA/US imaging begins. Because the number of channels is one-half of the number of US transducer elements, one B-mode PA/US image is acquired with two laser shots, and thus the PA imaging rate is 5 frames per second. At exactly the same time, when the scanner system receives the first trigger signal from the laser system, the scanner starts moving in the elevational direction, traversing the full scanning range *y_Scan_* during *T_Scan_*:

T_Scan_ = T_Image_ × n_Frame_

$$y_{Scan}=R\times \left(1-\text{c}\text{o}\text{s}\theta_{Scan}\right)$$

Where *T_Scan_* is the full scanning time, *T_Image_* is the imaging time per frame (0.2 s), *n_Frame_* is the number of scanned frames, R is the length of the arm, and $\theta_{Scan}$ is the full scanning angle between the arm and origin. *T_scan_*'s time range can be from milliseconds to seconds, depending on *n_Frame_*. The scanning range is maximized when the arm moves from the origin to the maximum angle of 180 degrees. Because the movement of the scanner is managed by the Scotch yoke mechanism, the distance of each frame depends on the angle θ:

$$\theta =\theta_{Scan}\times \frac{n}{n_{Frame}}$$

Where n is the current frame index. When each B-mode PA/US image is obtained with *T_Scan_*, *T_Image_*, θ_Scan_, the scanning index numbers of the previous frame $n_{i}$ and the subsequent frame $n_{f},$ the scanner is moved by y_Image_ (a one frame scanning range) according to the scanning angle of the previous frame $\theta_{i}$ and the subsequent frame $\theta_{f}$ as follows:

(2) $$y_{Image}=R\times |(1-\cos\theta_{i})-(1-\cos\theta_{f})|$$

$$=R\times \left|\text{c}\text{o}\text{s}\left(\theta_{Scan}\times \frac{n_{f}}{n_{Frame}}\right)-\left(\text{c}\text{o}\text{s}\left(\theta_{Scan}\times \frac{n_{i}}{n_{Frame}}\right)\right|\right)$$

To make the image dense, the scanning step size dy is adjusted through the step angle $\theta_{s\_i}$ and $\theta_{s\_f}$ as follows:

$$dy=R\times \left|\text{c}\text{o}\text{s}\theta_{s\_f}-\text{c}\text{o}\text{s}\theta_{s\_i}\right|$$

For spectral unmixing of multi-wavelength PA images, the scanning step size dy is divided by the number of laser wavelengths to be used. As the number of wavelengths increases, the scanning step size, dy, becomes dense and the full scanning time *T_Scan_* increases. To minimize motion artifacts while the transducer is being translated, we set dy (at most 0.63 mm) to be much less than the elevational beam width of the linear array US transducer (about 1 mm at the depth of 30 mm), which allows a single PA image to be safely acquired within the elevational resolution of the transducer. When the scanning is completed, the imaging probe automatically returns to the origin position (see Supplementary Video S2).

### 2.2 3D PA/US image processing

3D PA/US image processing is performed by online and offline processes. The online processes include real-time reconstruction and data storage. After receiving signals from the US transducer, real-time B-mode PA/US imaging is performed using a reconstruction algorithm that is accelerated by a graphics processing unit (GPU), and at the same time PA raw data and US beam-formed image data are stored [28,30,31]. The offline processes are carried out in three steps: (1) PA beamforming, (2) B-mode image stacking, and (3) volume data interpolation. The PA raw data is normalized by the laser output power and reconstructed into beam-formed image data by Fourier-domain beamforming [[32], [33], [34]]. The PA/US beam-formed image data for each frame are stacked in the elevational direction to create PA/US volume image data. Because dy is dependent on cosθ, the volume image is relatively dense at the beginning and the end of the scan range, as shown in Fig. 2b. Until this step, the stacked images are distorted by the non-uniform scanning because the position of each B-mode frame is represented as a digital index instead of the actual physical position. To reshape the images regarding the actual positions, we applied interpolation on the stack of images into a uniform grid where the grid index directly represents the actual position. This process enables representation of the images in the actual scales without being affected by the non-uniform acquisition. To obtain a uniform volume image, interpolation is performed in the elevational direction, with a uniform scanning step size $\Delta y=y_{Scan}/n_{Frame}.$

### 2.3 Performance evaluation via phantom experiments

**Fig. 3:**
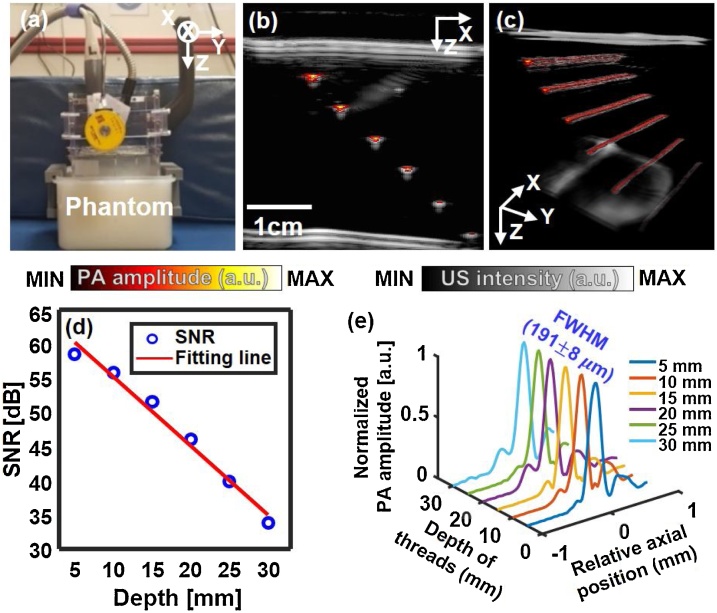
3D PA/US imaging of a tissue-mimicking phantom. (a) Photograph of the experimental setup. (b) Overlaid B-mode PA/US image. (c) Volume rendered PA/US image (see Supplementary Video S3). (d) Quantified SNRs of black threads at various depths. (e) Quantified axial thickness of threads at different depths, measured by FWHM. PA, photoacoustic; US, ultrasound; SNR, signal-to-noise ratio; and FWHM, full width at half maximum.

The system’s performance was evaluated through phantom experiments (Fig. 3). The scanner was positioned on a phantom (${\text{μ}}_{eff}:\;0.9$ cm^−1^) consisting of water, gelatin, and intralipid where ${\text{μ}}_{eff}$ is the effective attenuation coefficient, and the scanner was scanned along the elevation direction (i.e., y direction) (Fig. 3a) [18]. To identify the SNRs ​​along the depth, six black threads having a thickness of 90 μm were positioned as light absorbers at 5 mm intervals in the lateral and axial directions [9]. US gel (Ecosonic, SANIPIA, Republic of Korea) was applied to the scanner and phantom for acoustic coupling. The laser wavelength was 797 nm, and the pulse energy at the surface of the phantom was 7.5 mJ/cm^2^, which was below the American National Standards Institute (ANSI) safety limit of 31.3 mJ/cm^2^ at this wavelength. The *y_Scan_*, FOV, and *T_Scan_,* were 31.4 mm, 31.4 × 38.4 mm^2^, and 11.4 s, respectively.

### 2.4 *In vivo* 3D PA/US imaging of various human body parts

The suitability of the system for clinical translation was evaluated through *in vivo* human imaging experiments by imaging various body parts of four healthy volunteers, such as the neck, wrist, and thigh. The experiments were conducted according to protocols of the Institutional Review Board of Pohang University of Science and Technology (POSTECH). For acoustic coupling, US gel was used at the top and bottom of the water tank and examined body parts. We used the laser wavelength of 850 nm and the pulse energy of 6.1 mJ/cm^2^ at the skin, which was less than the ANSI safety limit of 39.9 mJ/cm^2^ at this wavelength. To prevent eye damage from the laser exposure, the volunteers and examiner wore laser safety goggles. The *y_Scan_*, FOV, and *T_Scan_* were 40.0 mm, 40.0 × 38.4 mm^2^, and 20.0 s, respectively. To enhance the visualization of vessels, we compensated the PA raw data using time gain compensation with an exponential function. After then, we performed depthwise sectioning of the PA volume and removed underlying noise signals by thresholding relatively to the maximum amplitude of each section. Further, we differentiated vessels located at shallow depths and deep depths using the MAP method. For shallow depth PA MAP images, we used 3.0–6.8, 2.0–6.8, and 3.6–9.4 mm depth data from the skin for the neck, wrist, and thigh, respectively. For deep depth PA MAP images, we used 6.8–11.6, 6.8–10.0, and 9.4–11.6 mm depth data from the skin for the neck, wrist, and thigh, respectively. For validating the PA vascular images, we obtained US Doppler images of the corresponding body parts using the same US transducer. Because our scanner holds the US transducer orthogonally to the skin, it is not appropriate for Doppler imaging which needs manual or electronic tilting of the transducer for better sensitivity. Thus, we detached the transducer from the scanner and obtained the Doppler images from the similar position in the scanned region.

### 2.5 Multi-wavelength PAI of a human instep

Multi-wavelength PAI was conducted using wavelengths of 775, 797, and 825 nm, which were chosen to be as evenly distributed as possible with respect to the isosbestic point (797 nm) of oxy-hemoglobin and deoxy-hemoglobin. While using more wavelengths would enhance the accuracy of spectral unmixing, only three wavelengths were selected to perform fast imaging acquisitions in clinical environments. We imaged the instep of a volunteer’s foot, by placing the scanner carefully on the instep to avoid any unwanted signals from hair or abnormal skin and applied US gel for acoustic coupling. Based on the fluence compensation method in [4], we estimated the light fluence by the mean background intensity at each depth in each reconstructed PA image, and normalized each PA image accordingly. To apply the method in our results, we first detected the contour of the skin using the 3D PHOVIS software [35], and segmented the depth at each B-mode PA image relatively to the detected skin position. To identify spectral trends at each wavelength, PA amplitude analysis of imaged areas #1, #2, #3 and #4 shown in Fig. 5b was performed. Each PA amplitude was normalized by calculating mean values of the imaged areas at each wavelength and dividing the values by the maximum mean value of them. Further, we applied the spectral unmixing on the PA volume data, and used maximum amplitude projection to map the corresponding relative sO_2_ values to the instep's vessel image. PA sO_2_ estimation method is described in Supplementary Note S1. The *y_Scan_*, FOV, and *T_Scan_,* were 31.4 mm, 31.4 × 38.4 mm^2^, and 34.2 s, respectively.

## 3 Results and discussion

### 3.1 Performance evaluation via phantom experiments

Fig. 3b and c show the overlaid B-mode PA/US image and volume rendered PA/US image (Supplementary Video S3) of the phantom, respectively, where the PA images are in a pseudo color and the US images in gray. All six black threads, located at depths up to 30 mm, are well visible, and US reverberation artifacts are also identifiable at the bottom of the images. PA and US thread images may look different in the axial and lateral direction, because log compression parameters are separately adjusted for each modality. Fig. 3d shows the linear decrease (10.1 dB / cm) in SNR (dB) along the depth direction, from which the 1/e decay penetration depth is calculated as 1.98 cm. Fig. 3e shows the PA axial profiles of the six threads at the different depths, with the calculated full width at half maximum (FWHM) values. The axial profiles are almost identical with the depth, and the FWHMs are 191 ± 8 μm. PA lateral, US axial and US lateral profiles are represented in Supplementary Fig. S1.

### 3.2 *In vivo* 3D PA/US imaging of various human body parts

**Fig. 4:**
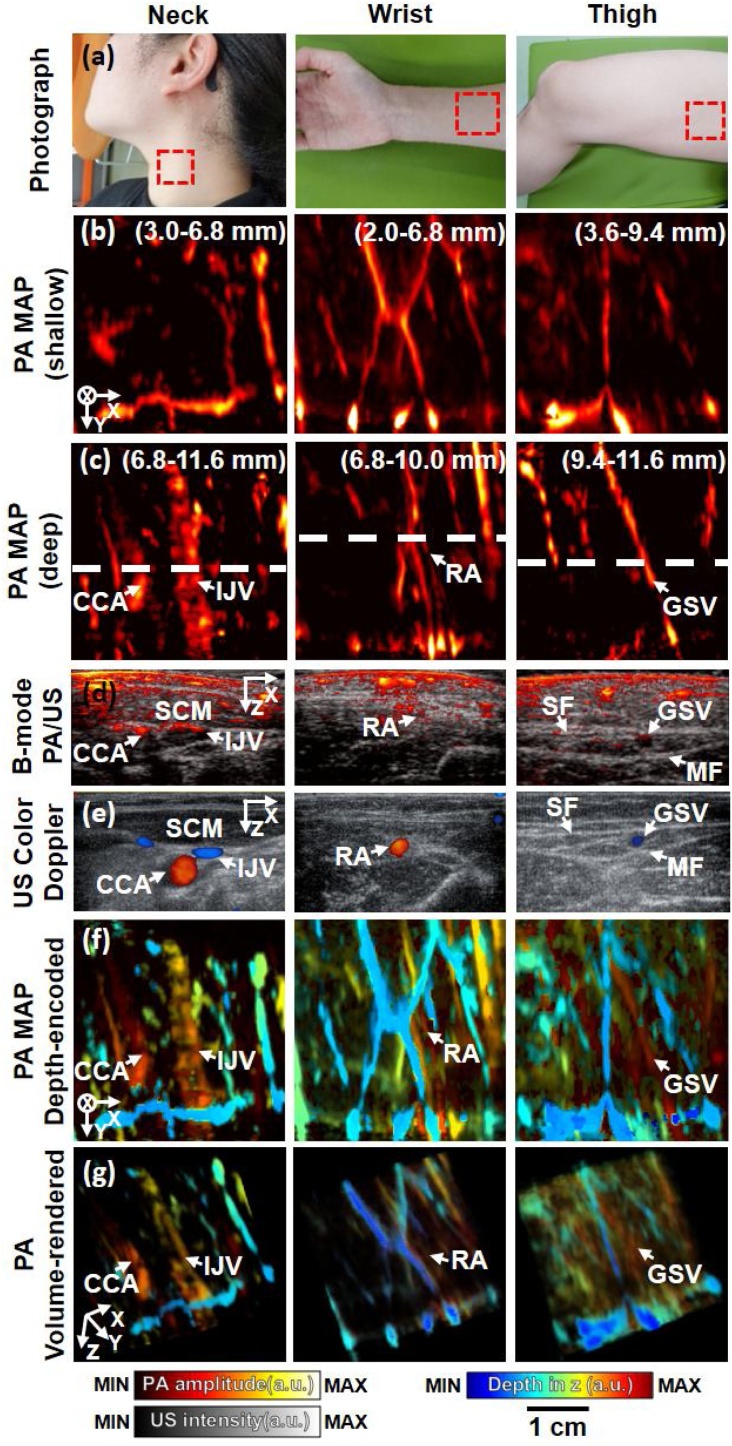
In vivo 3D PA/US imaging at various parts of a human body. (a) Photograph of the human imaging parts. (b) PA MAP images (shallow). (c) PA MAP images (deep). (d) Overlaid B-mode PA/US images. (e) US Color Doppler images. (f) PA MAP Depth-encoded images. (g) Volume rendered PA images (see Supplementary Video S4, S5, S6). PA, photoacoustic; US, ultrasound; and MAP, maximum amplitude projections; S, skin; SCM, sternocleidomastoid muscle; CCA, common carotid artery; IJV, internal jugular vein; RA, radial artery; SF, saphenous fascia; GSV, great saphenous vein, MF, muscular fascia.

The photograph in Fig. 4a shows imaged regions of the human body (e.g., neck, wrist, and thigh), with the imaged areas indicated by red dashed rectangles. Fig. 4b and c display the corresponding PA maximum amplitude projection (MAP) images, which show blood vessels located at shallow depths and deep depths (e.g., common carotid artery (CCA), internal jugular vein (IJV), radial artery (RA) and great saphenous vein (GSV)) of the neck, wrist, and thigh. The overlaid PA/US images are shown in Fig. 4d, cut along the white dashed lines in Fig. 4c. The US image acquired from the neck area shows the sternocleidomastoid muscle (SCM) and the CCA and IJV, which are located below the SCM, and the B-mode PA image shows the upper boundaries of the CCA and IJV. The RA is displayed in the both US and PA images. The GSV is shown in the thigh image, and it is surrounded by the saphenous fascia (SF) and muscular fascia (MF), which are represented in the US image, and the upper boundary of the GSV is shown in the PA image. We can confirm that the PA/US images of the CCA, IJV, RA, and GSV match well with the US color Doppler images (Fig. 4e). While US Doppler can better show the major vessels in cross-sectional views, the advantage of PA imaging comes from providing 3D PA vascular images using a linear array transducer and a mechanical scanner, which is difficult to obtain with the US Doppler imaging due to the scanner motion. As can be seen in Fig. 4b and c, PA images can well visualize the major vessels in a 3D view and also the small vessels which were not visible from the US Doppler images. The maximum PA imaging depths in the neck, wrist, and thigh were 9.8, 9.7, and 14.2 mm, respectively, found at the CCA, RA, and GSV, and its distributions in 3D space are shown in Fig. 4f, which are PA MAP depth encoded-images. Imaging depths diverse at each imaged part because of the different organization of body components, such as lipids, muscles, and melanin, that affects the speed of sound and optical/acoustic attenuation. Heterogeneous distributions of these body components affect the speed-of-sound distribution, which could degrade the beamforming process which typically assumes a constant speed of sound, and the acoustic/optical attenuation of the components might affect the detectable depth of the PA signals. Further, the presence of bone structures can block the acoustic wave propagation and light penetration. For instance, in our results in Fig. 4, the thigh had the best penetration depth of 14.2 mm because it consisted of mostly muscles, and it contained less melanin component on the skin. The wrist had the worst penetration depth of 9.7 mm because of the presence of bones such as radius and ulna [36]. The neck had relatively more lipid component and more melanin on the skin, which would have resulted in the penetration depth of 9.8 mm. Conservatively, we used thick PVC membranes to seal the system's water tank and prevent water leakage during the *in vivo* experiments, but acoustic attenuation in this material is relatively high. Low-density polyethylene (LDPE) may be a good alternative to increase the imaging depth, because its optical and acoustic attenuation is relatively small. In addition, we used 6.5 times less energy than the ANSI limit. We are using the maximum energy from the laser (about 120 mJ), which is delivered by fiber bundle with an efficiency of about 70 %. Then, the end of the fiber bundle is spread in a linear shape to make an illumination area of about 10 cm^2^. Furthermore, the PVC membrane layers attenuate the light delivery to the skin. As a result, our light fluence to the skin is far smaller than the limit value. As our next step, we are planning to enhance the light delivery by optimizing the beam shape and using a more transparent material for the membrane to enhance the penetration depth. Other than that, we used a linear array transducer having relatively high center frequency (∼ 8 MHz), thus using imaging probes with a lower frequency might increase the imaging depth. Fig. 4h shows volume-rendered PA images, in which blood vessels such as CCA, IJV, RA and GSV are clearly identified, as are vessels located at shallow depths (Supplementary Videos S4, S5, and S6) [35]. To improve the image quality, using the synthetic aperture focusing technique (SAFT) with a slit might improve the elevational resolution [37,38]. Motion artifacts can occur mainly due to the low frame rate, especially in the areas near the main body having many pulsational motions. To the best of our knowledge, there are two techniques to overcome this limitation. One is to use an electrocardiogram (ECG) sensor to compensate motion artifacts induced from heartbeats. By using ECG sensor, laser and non-heartbeat zone can be synchronized to remove motion artifacts. The other is to use cross-correlation methods between each image frame for compensating distance errors. As the next steps, we will develop algorithms using cross-correlation methods to reduce motion artifacts. The *in vivo* imaging results imply a versatile clinical translation of our handheld scanner. One promising clinical application is monitoring free flap transplantation, which embeds whole tissues that contain blood vessels in other areas with poor blood circulation. The most important aspect of this operation is the anastomosis of the blood vessels, so it is important to know the exact extent of the defect site [39]. The scanner can be conveniently used to image a variety of body parts and can view blood vessel distribution in 3D space, which can be useful for pre-operative and post-operative monitoring of the free flap.

### 3.3 Multi-wavelength PA imaging of the human instep

**Fig. 5:**
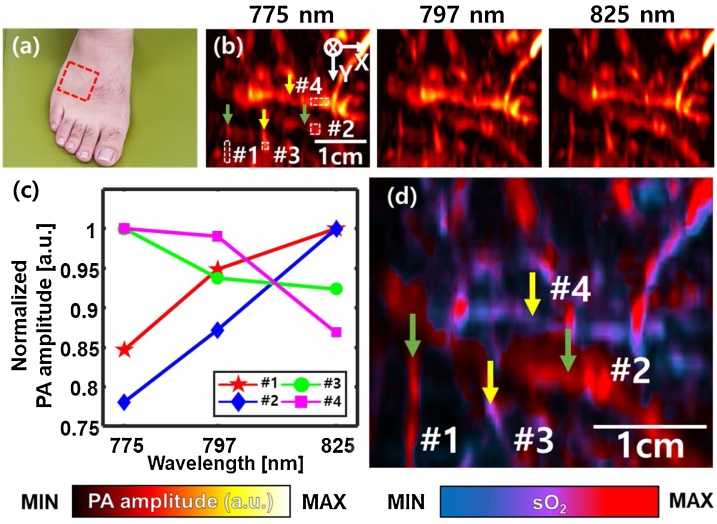
Multi-wavelength PA imaging of a human instep. (a) Photograph of the imaged human instep. (b) PA MAP images acquired at wavelengths of 775, 797, and 825 nm. (c) Normalized PA amplitudes of imaged areas represented by #1, #2, #3, and #4. (d) PA sO2 image. PA, photoacoustic; MAP, maximum amplitude projections; sO2, oxygen saturation.

The photograph of the human instep is shown in Fig. 5a, where the red dashed rectangle represents the imaged area. Fig. 5b shows PA MAP images for 775, 797, and 825 nm, respectively. Fig. 5c shows that the normalized PA amplitudes of imaged areas #1, #2, #3, and #4 in Fig. 5b. As the wavelength increases, the normalized PA amplitudes in the imaged areas #1 and #2 increase, while those in #3 and #4 decrease. Therefore, the blood vessels indicated by the green arrows can be regarded as containing relatively more oxy-hemoglobin than deoxy-hemoglobin, and the vessels indicated by the yellow arrows can be considered the opposite. These spectral trends obviously follow the molar extinction coefficients of oxy- and deoxy-hemoglobin at those wavelengths. The depths of the vessels can be confirmed via Supplementary Fig. S2, which shows PA MAP images in the XZ and YZ planes. Fig. 5d shows a multi-wavelength PA sO_2_ image, in which relatively higher and lower oxygenated blood vessels, indicated by the green and yellow arrows, are shown in red and blue. This sO_2_ information provided by the 3D PA/US imaging system can be useful in diagnosing various types of malignant cancer, because a malignant tumor is characterized by hypoxia, and 3D imaging can also be advantageous in distinguishing the margins of cancer areas. The scanner system can be used to further distinguish endogenous contrast agents such as melanin, which can be particularly beneficial in the diagnosis of the melanomas [40].

## 4 Conclusion

We presented a 3D clinical handheld PA/US scanner that uses a Scotch yoke mechanism, and we investigated its potential clinical use by performing *in-vivo* human imaging experiments. Acceptable image qualities were confirmed to the depth of 14.2 mm, and the convenience of the scanner was also proved by imaging various human body parts (e.g., the neck, wrist, thigh, and instep) that were difficult to image with previous imaging systems. Spectral unmixing was applied to provide sO_2_ information. Although technical developments such as membrane optimization and the use of a synthetic aperture with a slit are planned as next steps for better PAI, we believe that the currently implemented scanner can be applied to image such conditions of clinical interest as the free flaps, kidney failures, and diabetic feet. The scanner may be particularly advantageous for cancer imaging, where sO_2_ information is important. Other types of US transducers (e.g., convex or phased) can be also combined with the scanner to greatly enhance its clinical translatability.

## Declaration of Competing Interest

Chulhong Kim has financial interests in OPTICHO, however, did not support this research.
