# Supplementary material for: Three-dimensional clinical handheld photoacoustic/ultrasound scanner
Source: Photoacoustics. 2020 Mar 11;18:100173. doi: 10.1016/j.pacs.2020.100173 (PMC7090348; doi:10.1016/j.pacs.2020.100173)
Supplement: Supplementary file 1 [file mmc1.docx]

**Supplementary information for:**

**Manuscript number: PACS_D_19-00048R1**

**Manuscript title: Three-dimensional Clinical Handheld Photoacoustic/Ultrasound Scanner**

Changyeop Lee^§^, Wonseok Choi^§^, Jeesu Kim, and Chulhong Kim*

Department of Mechanical, Electrical, and Creative IT Engineering, Pohang University of Science and Technology (POSTECH), Pohang, Republic of Korea 37673

^§^These authors contributed equally on this work.

***Corresponding authors:**[chulhong@postech.edu](mailto:chulhong@postech.edu)

**Table of contents**

**Supplementary Figures**

Fig. S1. PA lateral, US axial and US lateral profiles of threads in the phantom1

Fig. S2. PA MAP images of the human foot in the XZ and YZ plane2

**Supplementary Note**

Note S1. PA sO_2_ estimation method3


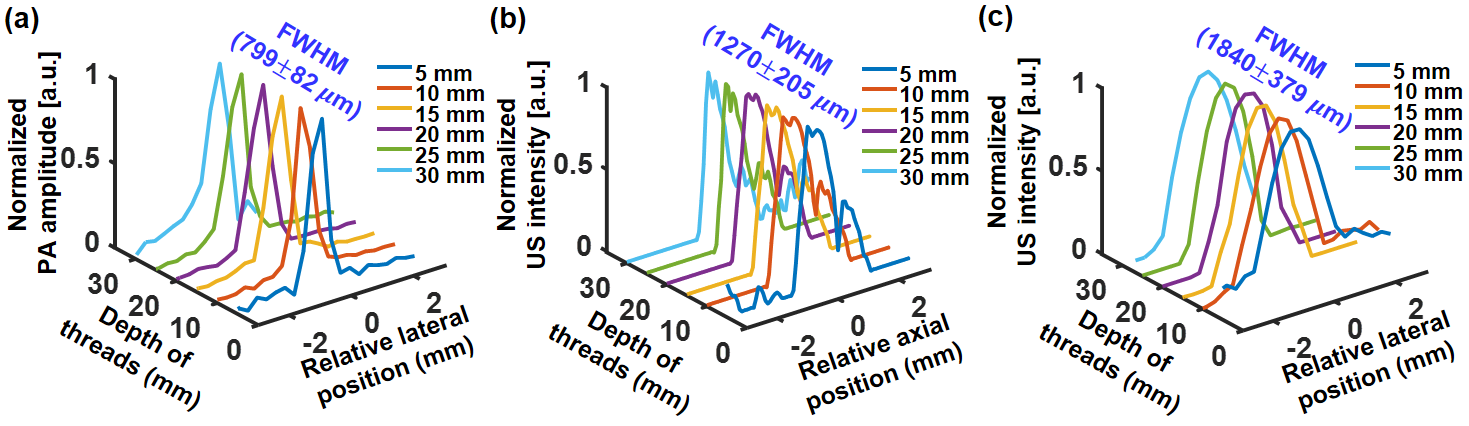


**Supplementary Fig. S1.** (a) PA lateral profile of threads at different depths measured by FWHM. (b) US axial profile of the log-compressed US image of threads at different depths measured by FWHM. (c) US lateral profile of the log-compressed US image of threads at different depths measured by FWHM. PA, photoacoustic; US, ultrasound; and FWHM, full width at half maximum.


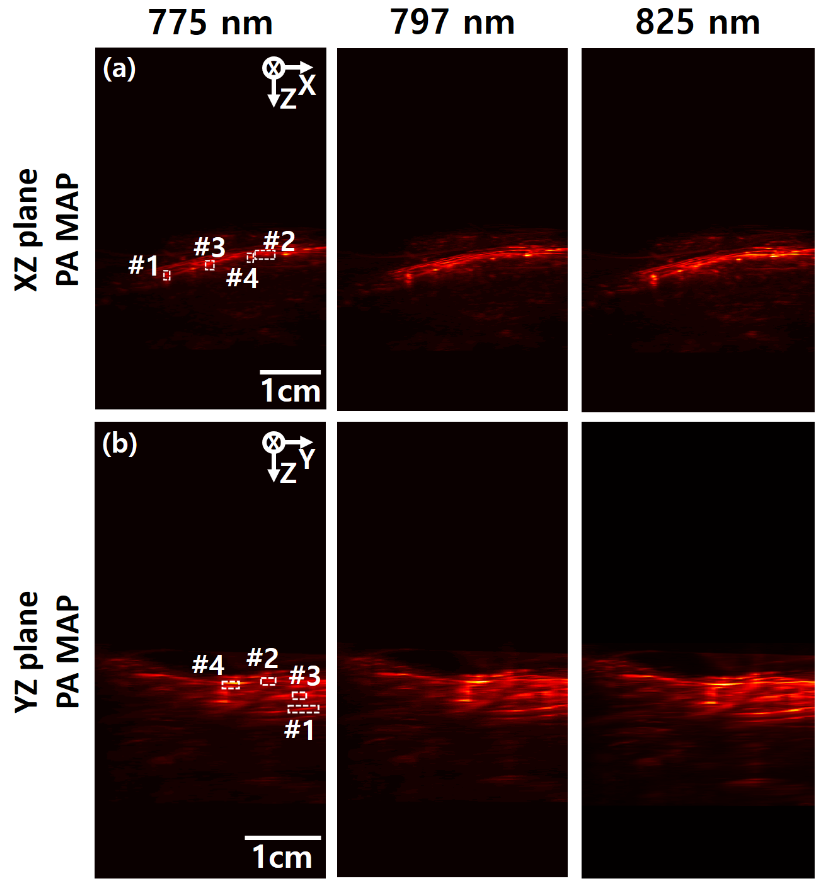


**Supplementary Fig. S2.** (a) PA MAP images in the XZ plane acquired at the representative wavelengths (e.g., 775, 797, and 825 nm). (b) PA MAP images in the YZ plane acquired at the representative wavelengths. White dashed rectangles indicate location of the vessels. Each depth of the blood vessels indicated by #1, #2, #3 and #4 is 1.5, 1.2, 1.5 and 1.9 mm, respectively. PA, photoacoustic; and MAP, maximum amplitude projection.

**Supplementary Note S1.**

*PA amplitude at wavelength* $\lambda_{i}$ *is proportional to the light fluence and the optical absorption, i.e.,* $A\left( \lambda_{i} \right)\propto F\left( \lambda_{i} \right)\cdot\mu(\lambda_{i})$*, where* $A\left( \lambda_{i} \right)$ *is the measured PA amplitude,* $F\left( \lambda_{i} \right)$ *is the light fluence, and* $\mu(\lambda_{i})$ *is the optical absorption coefficient at wavelength* $\lambda_{i}$*, respectively. For accurate calculation of spectral unmixing, the light fluence must be properly estimated and compensated. Based on the fluence compensation method in [4], we estimated the light fluence by the mean background intensity at each depth in each reconstructed PA image, and normalized each PA image accordingly. To apply the method in our results, we first detected the contour of the skin using the 3D PHOVIS software [35], and segmented the depth at each B-mode PA image relatively to the detected skin position.*

*After normalizing with the optical fluence, PA sO_2_ values can be estimated from the following equation.*

$$P=\left[ \begin{matrix} p\left( \lambda_{1} \right) \\ p\left( \lambda_{2} \right) \\ \vdots\\ p\left( \lambda_{N} \right) \end{matrix} \right]=\left[ \begin{matrix} \epsilon_{HbO_{2}}\left( \lambda_{1} \right) & \epsilon_{Hb}\left( \lambda_{1} \right) \\ \epsilon_{HbO_{2}}\left( \lambda_{2} \right) & \epsilon_{Hb}\left( \lambda_{2} \right) \\ \vdots& \vdots\\ \epsilon_{HbO_{2}}\left( \lambda_{N} \right) & \epsilon_{Hb}\left( \lambda_{N} \right) \end{matrix} \right]\left[ \begin{matrix} C_{HbO_{2}} \\ C_{Hb} \end{matrix} \right]=MC$$

*Here,* $p(\lambda_{i})$ *is the fluence-normalized PA amplitude at wavelength* $\lambda_{i}$*,* $\epsilon_{HbO2}(\lambda_{i})$ *and* $\epsilon_{Hb}(\lambda_{i})$ *are the molar extinction coefficients of HbO_2_ and Hb, respectively, at wavelength* $\lambda_{i}$*, and* $C_{HbO_{2}}$ *and* $C_{Hb}$ *are the relative concentrations of HbO_2_ and Hb, respectively. Using pseudo-inverse of* $M$ *on both sides of the equation, we can get the estimation of the concentrations as* $\hat{C}=\left( M^{H}M \right)^{-1}M^{H}P$*. From this, we can calculate the sO_2_ as* $C_{HbO_{2}}$*/*${(C}_{HbO_{2}}+C_{Hb}).$
